# Supplementary material for: Structure of a stripped-down and tuned-up far-red phycobilisome
Source: Commun Biol. 2025 Jun 10;8:907. doi: 10.1038/s42003-025-08326-y (PMC12152189; doi:10.1038/s42003-025-08326-y)
Supplement: Supplementary file 1 — Supplementary Material [file 42003_2025_8326_MOESM1_ESM.pdf]

Structure of a stripped-down and tuned-up far-red phycobilisome

Giovanni Consoli[1†], Ho Fong Leong[1†], Geoffry A. Davis, [1§†], Tom Richardson[1], Aiysha McInnes[1], James W. Murray[1], Andrea Fantuzzi[1]*, A. William Rutherford[1]*

[1]Department of Life Sciences, Imperial College; London, SW7 2AZ, United Kingdom.

*Corresponding author. Email: a.rutherford@imperial.ac.uk, a.fantuzzi@imperial.ac.uk

†These authors contributed equally to this work

§Present address: Department of Plant Biochemistry, Biology, Ludwig Maximilian University of Munich; Planegg-Martinsried, 82152, Germany.

Supplementary Figures:

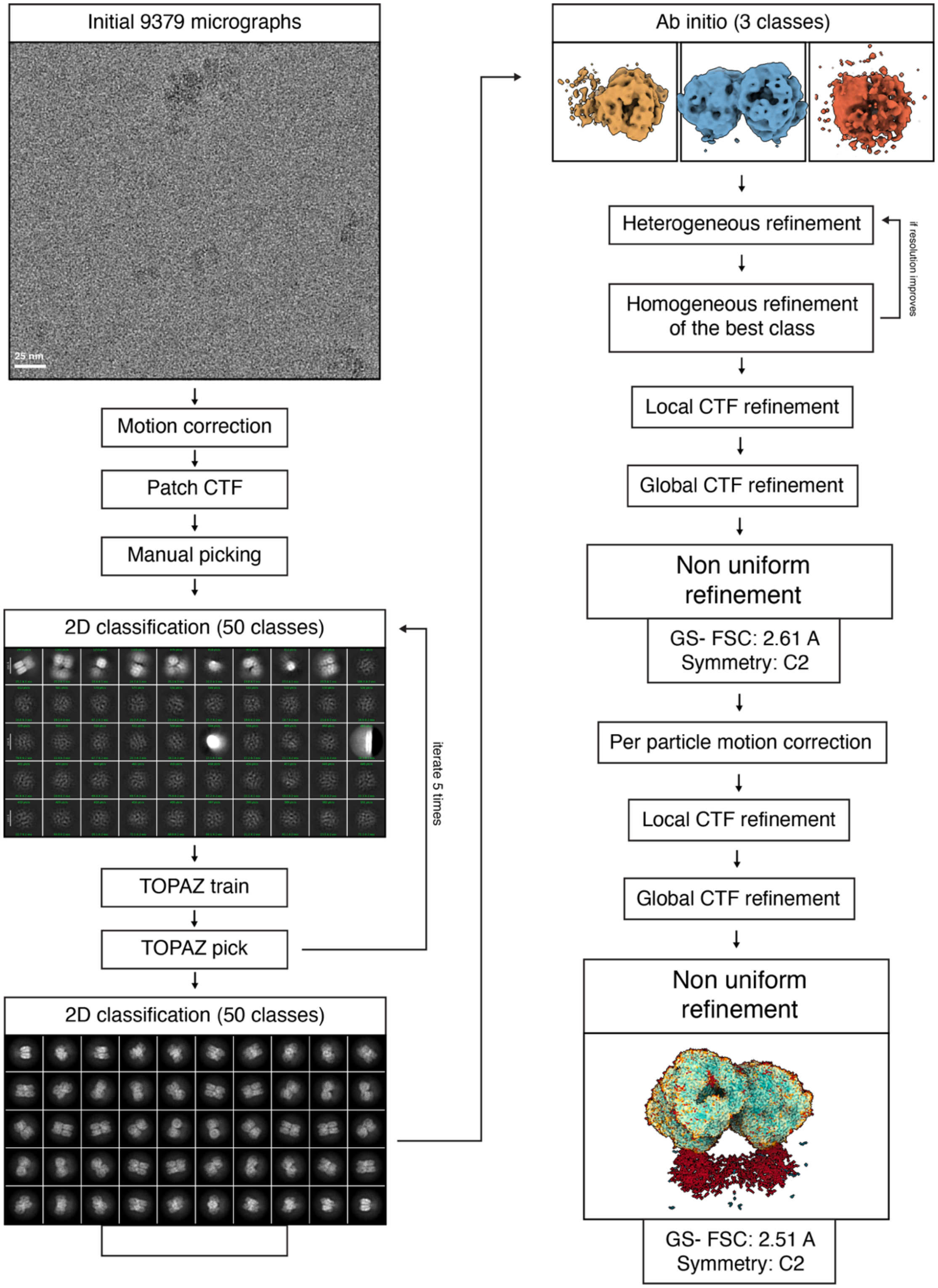

Supplementary Figure 1 - CryoSPARC workflow for the SPA structure determination of the FR-APC complex

CryoSPARC workflow for the determination of the map of the FR-APC complex.

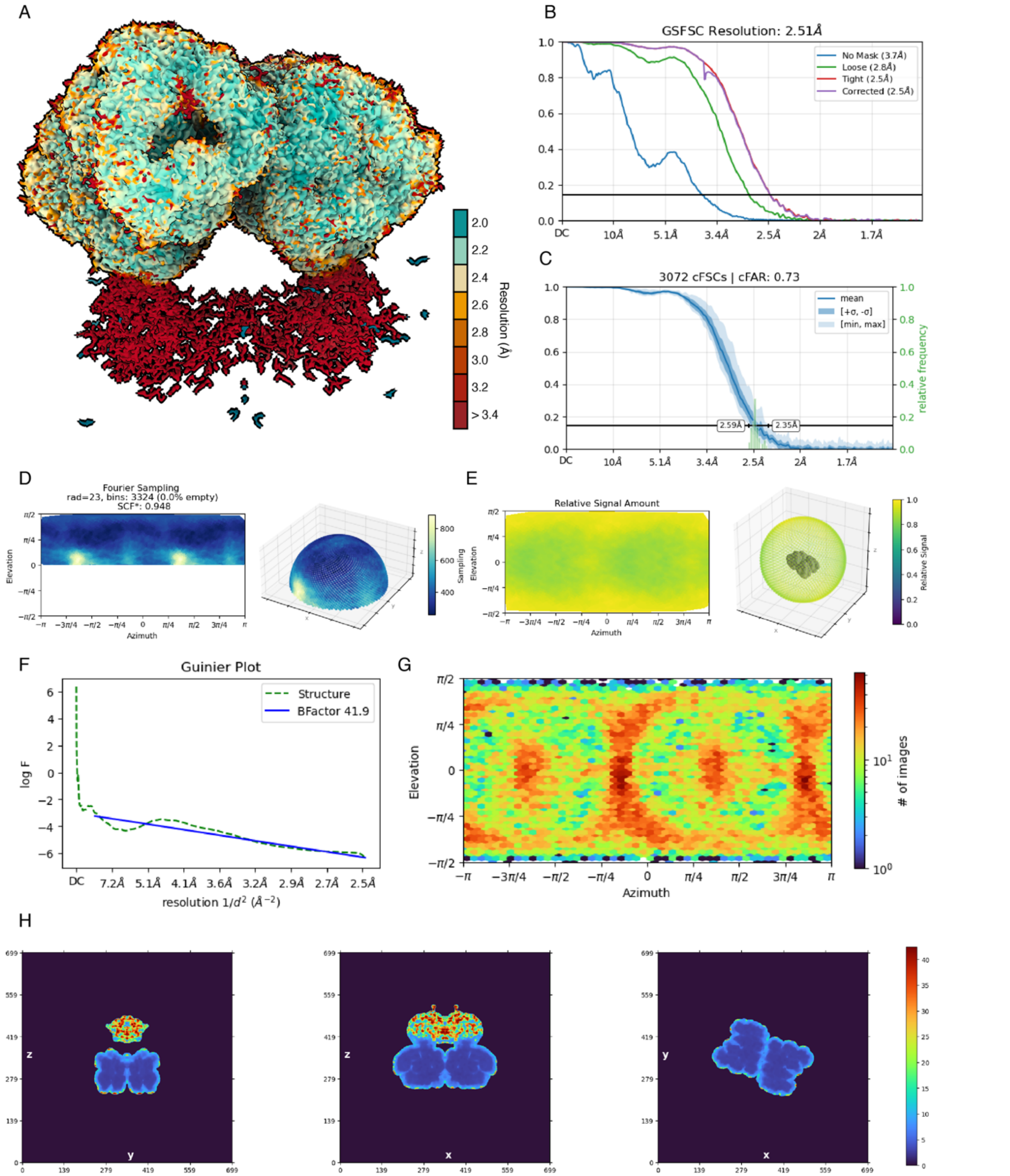

Supplementary Figure 2 - CryoSPARC workflow for the SPA structure determination of the FR-APC complex

A) Local resolution map of the FR-APC complex colored according to the colorbar on the side. B) GS-FSC plot of the resolution of the complex. C) cFSC plot of the resolution of the complex. D) Fourier sampling of the final reconstruction. E) Plot of the relative signal vs the viewing direction obtained with Cryosparc's orientation diagnostics tools. F) Guinier's plot of the FR-APC complex. G) Viewing direction distribution. H) Local resolution map at FSC = 0.5.

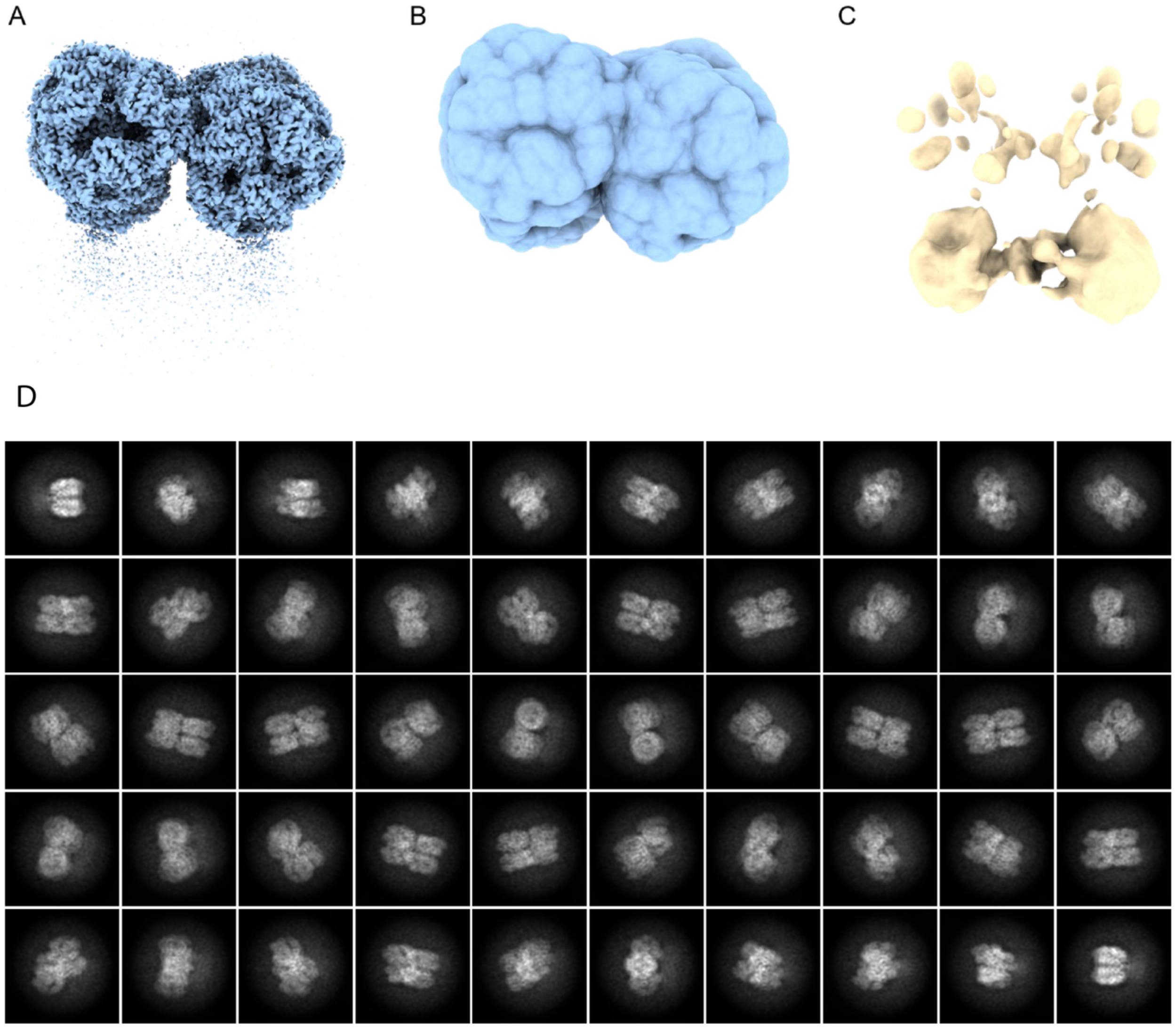

Supplementary Figure 3 – Local refinement mask and results.

A) Original map. B) Mask used to subtract from the particle set that covers the FR-APC cylinders. C) Example of a local refined map at the putative Photosystem II region showing only featureless blobs. To try and resolve the low-quality density that was observed on the membrane-side of FR-APC, multiple rounds of local refinements with different masks, with and without symmetry, were done. Briefly, masks of the APC cylinders were created using segger in ChimeraX. They were padded, dilated, and used to subtract the APC cylinders density from the particle set (symmetry expanded where appropriate) in a particle subtraction job in CryoSPARC (Fig. S5B). The resultant particle set was used to reconstruct an initial density, which was then fed into local refinement jobs. Unfortunately, none of the attempts were fruitful in resolving the putative Photosystem II density. Only featureless blobs were observed. D) 2D classes are presented here enlarged to show the presence of the local increase in density where PSII is expected to bind.

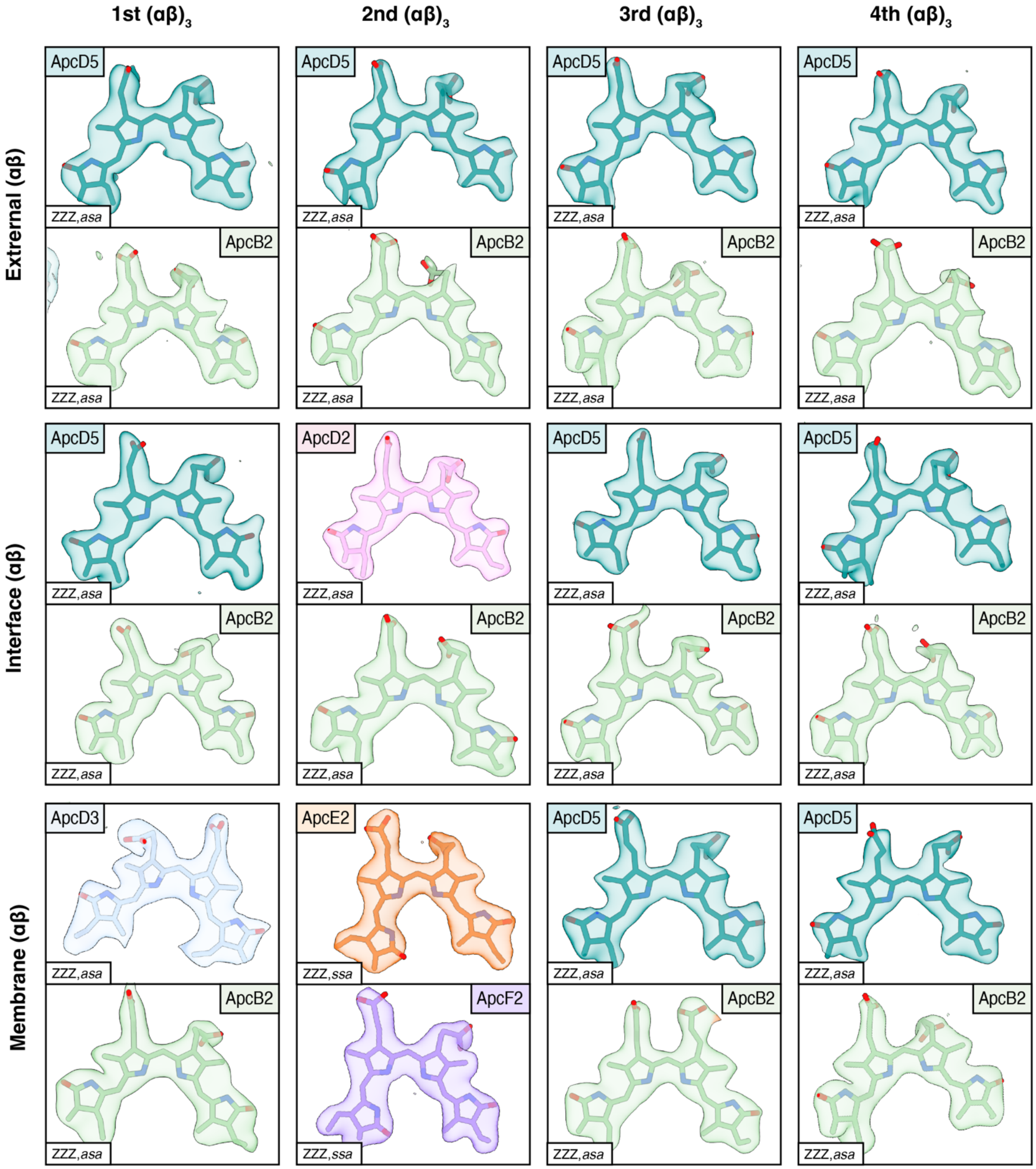

Supplementary Figure 4 – ESP density of each of the CYC pigments present in the FR-APC complex

ESP density of the bilin pigments discussed in the paper. The ESP density and the bilin models are represented in the same colors used throughout the main text, each column of pigments represents the bilins contained in an alpha beta trimer, as represented in Fig. 1, the 3 rows, composed of 2 bilins each, represent each a set of 4 monomers aligned with respect to the position of the complex and of the membrane.

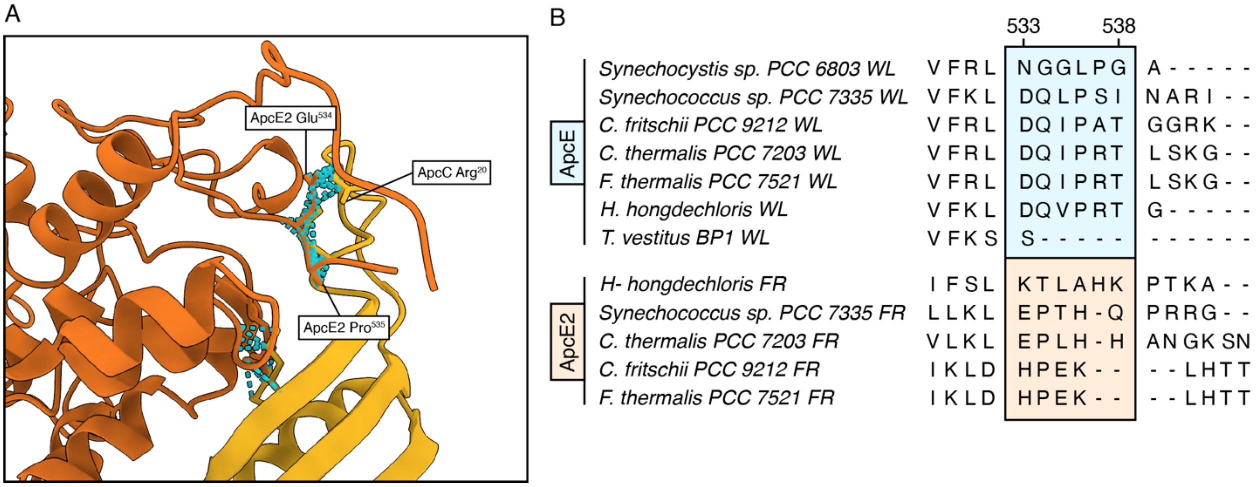

Supplementary Figure 5 - Clashes between distal ApcC and ApcE2

A) Structural analysis of the potential interaction between ApcC and ApcE2 if ApcC was in the same position compared to WL-PBS. ApcE2 is represented in orange and ApcC is represented in yellow, in blue are represented the molecular clashes between the two subunits B) phylogenetic analysis of the ApcE and ApcE2 FR and WL. In blue and orange, the areas of non-conservation in the C-terminal are highlighted.

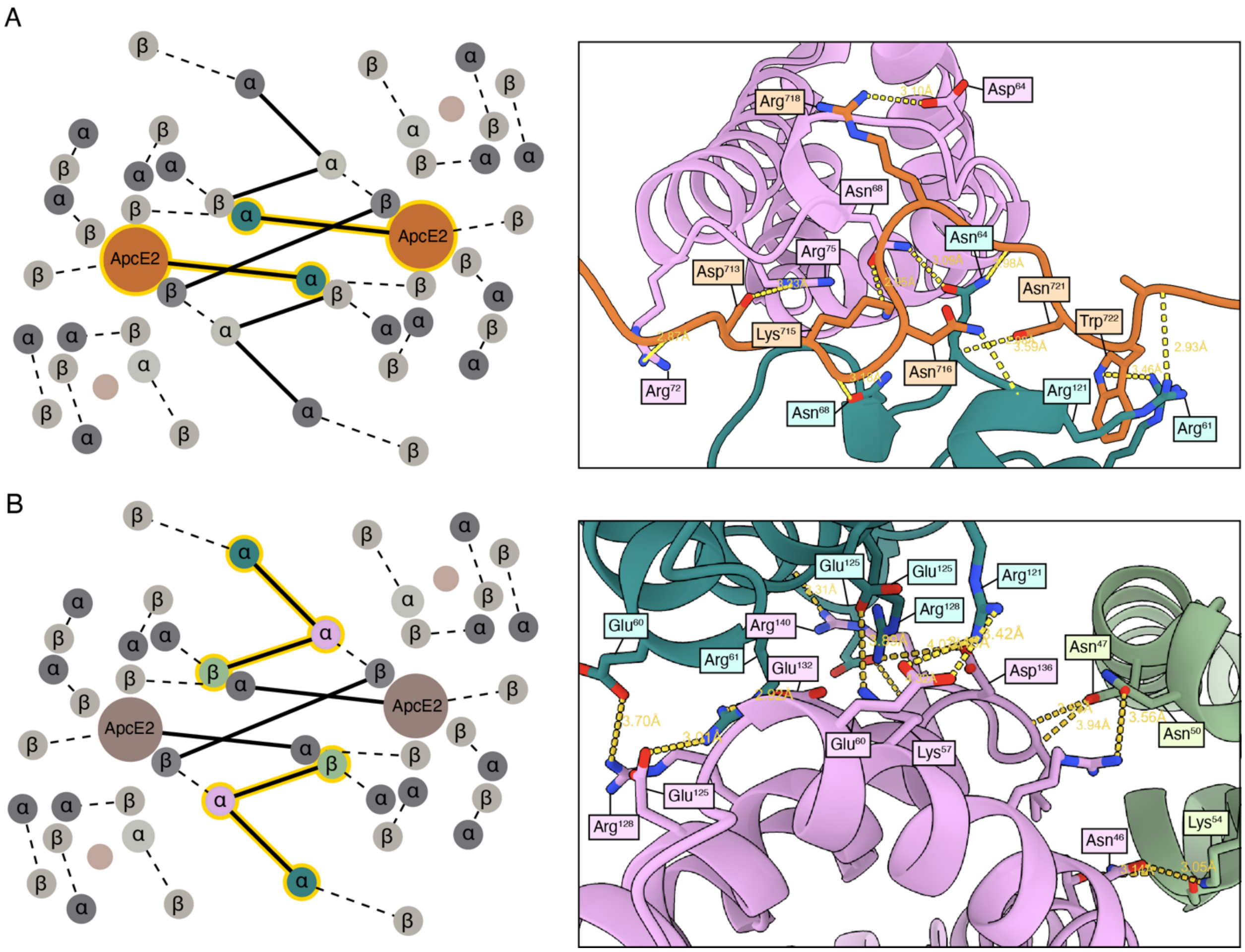

Supplementary Figure 6 – Interactions at the dimer interface between the two FR-APC cylinders.

A) The interaction of ApcE2 (in orange), with an ApcD5 (in teal) subunit of the third ring of the opposing cylinder are analyzed on the panel on the right. B) ApcD2 (in pink) interactions with an ApcB2 (green) of the third ring and an ApcD5 (in teal) subunit of the fourth ring of the opposing cylinder are analyzed in the panel on the right. On the left of panel A and B the network of the interaction between monomers of the bicylindrical FR-APC is represented. Subunits are represented as circles of dimension proportional to their length in amino acids. Interaction of α and β subunits to form (αβ) monomers are represented as dashed lines, while inter-cylinder interaction are represented by black solid lines.
